# Ten simple rules for creating a brand-new virtual academic meeting (even amid a pandemic)

**DOI:** 10.1371/journal.pcbi.1008485

**Published:** 2020-12-18

**Authors:** Scott Rich, Andreea O. Diaconescu, John D. Griffiths, Milad Lankarany

**Affiliations:** 1 Division of Clinical and Computational Neuroscience, Krembil Research Institute, University Health Network (UHN), Toronto, Ontario, Canada; 2 Krembil Centre for Neuroinformatics, Centre for Addiction and Mental Health, Toronto, Canada; 3 Department of Psychiatry, University of Toronto, Toronto, Ontario, Canada; 4 KITE Research Institute, Toronto Rehabilitation Institute, UHN, Toronto, Ontario, Canada; 5 Institute of Biomedical Engineering, University of Toronto, Toronto, Ontario, Canada; Dassault Systemes BIOVIA, UNITED STATES

## Abstract

The increased democratization of the creation, implementation, and attendance of academic conferences has been a serendipitous benefit of the movement toward virtual meetings. The Coronavirus Disease 2019 (COVID-19) pandemic has accelerated the transition to online conferences and, in parallel, their democratization, by necessity. This manifests not just in the mitigation of barriers to attending traditional physical conferences but also in the presentation of new, and more importantly attainable, opportunities for young scientists to carve out a niche in the landscape of academic meetings. Here, we describe an early “proof of principle” of this democratizing power via our experience organizing the Canadian Computational Neuroscience Spotlight (CCNS; crowdcast.io/e/CCNS), a free 2-day virtual meeting that was built entirely amid the pandemic using only virtual tools. While our experience was unique considering the obstacles faced in creating a conference during a pandemic, this was not the only factor differentiating both our experience and the resulting meeting from other contemporary online conferences. Specifically, CCNS was crafted entirely by early career researchers (ECRs) without any sponsors or partners, advertised primarily using social media and “word of mouth,” and designed specifically to highlight and engage trainees. From this experience, we have distilled “10 simple rules” as a blueprint for the design of new virtual academic meetings, especially in the absence of institutional support or partnerships, in this unprecedented environment. By highlighting the lessons learned in implementing our meeting under these arduous circumstances, we hope to encourage other young scientists to embrace this challenge, which would serve as a critical next step in further democratizing academic meetings.

## Introduction

Of the myriad lessons learned by the academic community in the age of the Coronavirus Disease 2019 (COVID-19), perhaps the most salient has been the reminder of how quickly our lives and routines are subject to change. Indeed, the coronavirus has swiftly and significantly disrupted nearly all aspects of how scientific research is performed and disseminated. However, the academic community may have found some silver linings during these unprecedented times in the opportunities provided by the virtual domain.

Perhaps the most challenging aspect of academia to transition into this setting is the academic meeting. Conferences, workshops, and the like are (traditionally, at least) central to the communication, critique, and constructive development of new knowledge and understanding. They are also the locus and medium of most international collaborative activity and provide the social and interpersonal “glue” necessary for such collaborations to be successful. Unfortunately, these events were among the first parts of academic research to be directly affected by the pandemic, with many such meetings being delayed or canceled outright [1]. As the realities of the pandemic came into focus, many more conferences announced that they would transition in some form to the virtual setting.

Interestingly, the first meetings to test this new virtual setting were not so-called legacy conferences (i.e., those that have existed for years, typically associated with a particular professional association or institution), but rather new conferences tailored specifically for this environment [2,3]. Our creation and implementation of the “Canadian Computational Neuroscience Spotlight (CCNS)” is an illustrative case study of these early forays. Spurred by the cancelation of the yearly Canadian Association for Neuroscience meeting, and the indefinite postponement of a planned symposium highlighting the growing computational neuroscience community in Toronto, we decided to create an entirely new meeting to fill this void left on our academic community’s calendar. Starting a meeting from scratch allowed us to create something unique, rather than simply porting a physical conference into the digital space. The result was a “trainee-focused” meeting, considering that it is these young scientists who will likely be most affected by the loss of the presentation and networking opportunities that conferences typically provide [4].

**Fig 1:**
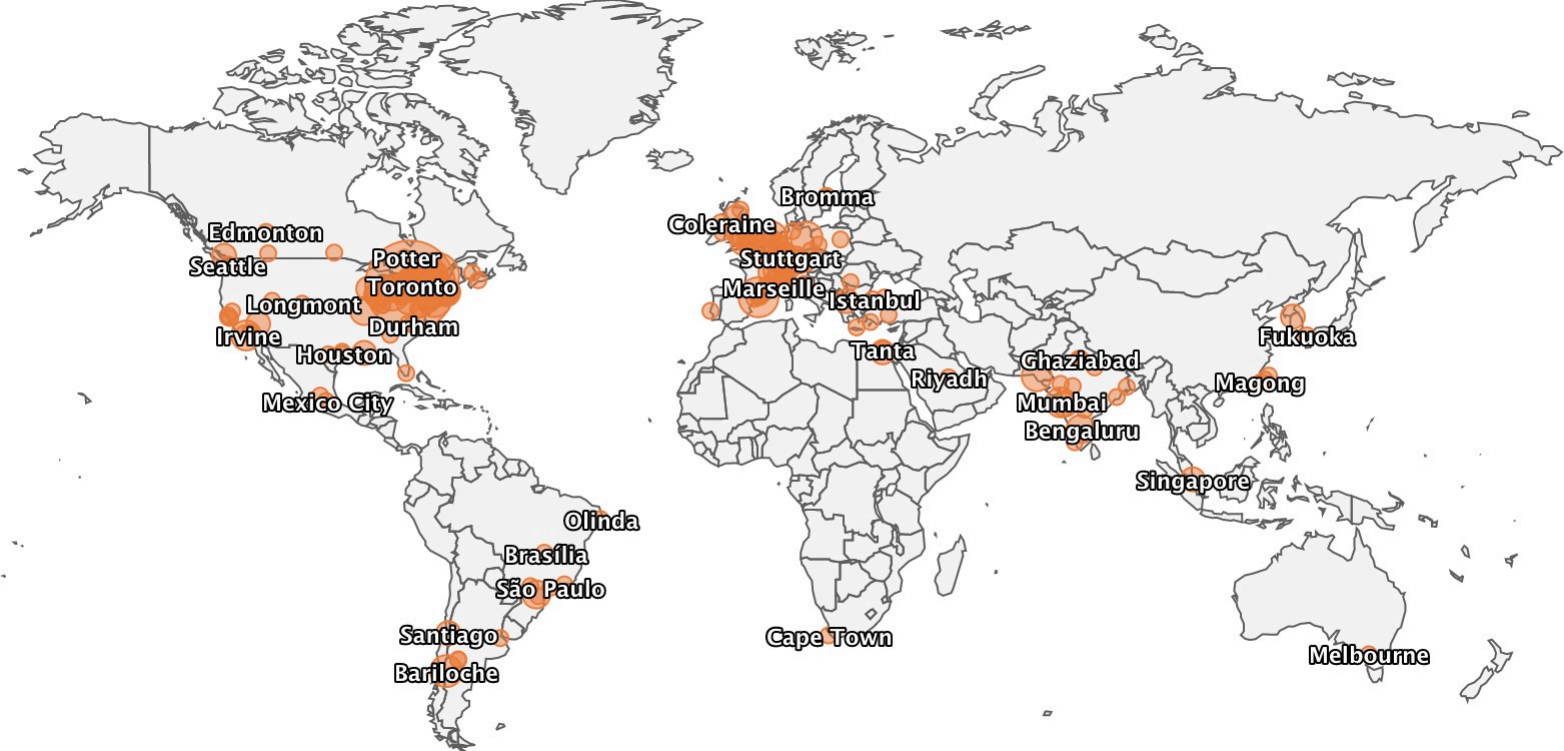
A map of the locations of the registrants for the Canadian Computational Neuroscience Spotlight, generated via the Crowdcast.io platform.

CCNS was successful well beyond our initial goals (which were approximately 100 primarily local participants, the expected attendance at the postponed Toronto symposium). Our meeting had more than 450 international registrants (including representation from every continent; see Fig 1), with dozens of additional registrations coming after the meeting in order to view sessions on “replay.” While our meeting placed a “spotlight” on the Canadian computational neuroscience community, our invited speakers and trainee presenters were similarly international. This was all the more exciting considering that the idea of CCNS did not exist before the pandemic: The conceptualization, planning, and implementation of this meeting all happened in approximately 10 weeks and entirely using virtual tools.

With the pandemic continuing to evolve, it is clear that academic conferences must as well. However, the success of CCNS shows that this evolution can be for the better and have a “democratizing” effect on not only the attendance at, but also the creation and planning of, academic meetings. We believe that many upcoming online meetings can learn from our experience, with the following “10 simple rules” for creating a brand-new virtual academic meeting serving as a useful blueprint for this endeavor.

## Rule 1: Remember that organizing an online meeting can be done at any career stage

The CCNS organizing committee was made up of 1 postdoc (Scott Rich) and 3 traditionally defined “early career researchers (ECRs)” (Andreea O. Diaconescu, John D. Griffiths, and Milad Lankarany). This factor made CCNS distinct from many of the other new conferences to arise in the preceding few months, many of which were organized by senior faculty. With CCNS serving as a pioneering “proof of concept” that brand-new academic meetings can be spearheaded by young academics, this development has the potential to help broaden and diversify who organizes conferences and, in turn, who is given the platform provided by such meetings.

## Rule 2: Recognize that the online setting eliminates institutional support as a barrier for entry

Not only was CCNS planned entirely by postdocs and ECRs, but the meeting was also not sponsored by or partnered with any academic institutions or journals. This too made CCNS distinct from other new online meetings, many of which were officially sponsored by a university or academic journal, which facilitates both the advertising and financial elements of planning such a meeting. This should serve as further encouragement for anyone wishing to claim a space in the new virtual conference frontier; such partnerships are no longer essential, with many of the logistical and bureaucratic burdens of conference planning eliminated in the digital setting.

## Rule 3: Utilize the online setting to accelerate planning and sidestep logistical challenges

In-person conferences typically require significant effort in organizing a physical space to host hundreds of people, independent of the effort required to plan the scientific aspect of the conference. This translates not only to an increased time commitment, but also to an increase in costs, both to secure a location and potentially to hire administrative assistance to oversee this aspect of the conference. Oftentimes, this motivates the need to secure partnerships with academic institutions, academic journals, or industry partners to help obtain these resources (as discussed above).

Virtual meetings do not face these specific organizational challenges, greatly accelerating the pace at which such meetings can be designed and implemented. Additionally, by sidestepping these logistical obstacles, online meetings minimize or eliminate any associated costs. In the case of CCNS, our costs were a pittance compared to in-person conferences (discussed in detail below), and the conceptualization and planning of CCNS took place in a matter of weeks, rather than months.

However, it bears acknowledging that communication is more challenging in the virtual setting; indeed, such communication is inherently easier with all the organizers in the same physical location. With this in mind, it is vital that prospective conference organizers make a clear plan to maintain constant communication during an online meeting. During CCNS, the 4 organizers were in constant communication using instant messaging tools. All the organizers were available at a moment’s notice to troubleshoot potential technical hiccups and provided constant real-time updates regarding the individual tasks to be performed. For example, using a Slack channel, the organizers were all immediately made aware of when each stage of a speaker’s setup (getting them into their session, initiating screen sharing, and finally being prepared for the session to begin) was completed. This was invaluable to ensuring that the conference proceeded smoothly regardless of any “behind the scenes” hurdles.

## Rule 4: Pick an online platform that emphasizes interactivity and learn its ins and outs

A significant portion of the planning of CCNS involved choosing, and then learning how to best utilize, a platform to host the event. While there are a handful of viable options, we chose the Crowdcast platform (Crowdcast.io). Not only was this platform cost-effective (see further discussion on pricing below), but we also felt that it best facilitated a level of interactivity approximating that seen at a traditional conference. Some useful features of this platform included the ability to have a continuous “chat” alongside of the presentation, a designated space to ask questions where others could “up-vote” questions that they most wanted to see answered, and the availability of talk “replays” that were automatically generated at the end of each session. Importantly, Crowdcast allows for these replays to be hosted on their platform indefinitely for a modest fee and thus remain available to the general public; indeed, we have seen a few dozen “late registrations” to the conference in order to view these replays. The entire set of CCNS sessions can be found at crowdcast.io/e/CCNS. Crowdcast also gives full “ownership” of the content to the creator, and as such, we have downloaded raw video files of all of our sessions and will be able to make them public via an alternative hosting platform if necessary.

Whether other virtual conferences choose to use Crowdcast or a competitor (such as the now ubiquitous Zoom or newer programs by tech giants like Google) is a matter both of personal preference and cost–benefit analysis. Regardless of the choice, it is crucial that the organizers of any online meeting be sufficiently fluent in the intricacies of that platform. Prior to CCNS, we familiarized ourselves with Crowdcast by using it to host regular lab meetings, most of our organizing committee meetings, and multiple “walkthroughs” of the CCNS itinerary. This allowed us to nimbly adjust to the inevitable technical glitches that arose during the meeting itself, having seen many of them during our practice sessions. We also offered all of our prospective speakers the opportunity to do a “test run” of their talks directly in the Crowdcast platform. This high comfort level was vital; it was clear that our intricate understanding of Crowdcast and the fact that most speakers had seen the platform in some form prior to the meeting proper eased some of our speakers’ anxieties, especially considering many of them were presenting online for the first time.

## Rule 5: Pass the savings facilitated by the digital platform on to attendees

Perhaps the most onerous hurdle to attending traditional, in-person conferences is the cost. Indeed, it is not uncommon for the combination of travel, accommodations, and registration fees to sum to thousands of dollars for large meetings. For young researchers who have yet to secure significant funding sources, or those located in areas that make travel additionally challenging, this often makes attendance at these meetings an untenable proposition [5]. Moreover, physical meetings already pose additional challenges, beyond the obvious financial ones, to those with health problems for which accessibility is a major concern [6]. Our success is further evidence that such barriers can be scaled by virtual conferences. The total costs for CCNS were limited to the short-term subscription to Crowdcast, which amounted to a few hundred dollars, allowing us to make our meeting completely free to attend; the goal of a free-to-attend meeting is likely obtainable for most online conferences at a similar scale.

## Rule 6: Exploit social media and other alternative forms of advertising in the absence of “name recognition”

For better or worse, there has historically been minimal competition when choosing conferences to attend; academic disciplines typically have a small number of associated professional societies that traditionally host yearly meetings. These conferences rely on this “name recognition” for consistent attendance and have existing platforms through which to advertise. CCNS lacked any of these tools. Instead, CCNS obtained its worldwide audience of more than 450 registrants entirely through social media, advertising via existing “listservs,” and word of mouth. (Unfortunately, Crowdcast does not at present provide further details regarding the number or location of live attendees at each session, but this is apparently a focus for potential improvements of the platform.) Moreover, more than 1,500 individuals were aware enough of the meeting to visit our registration homepage. This is proof that the ever-evolving social media landscape can be utilized to generate organic interest in new online meetings, independent of whether the meeting has a built-in audience.

## Rule 7: Embrace the opportunity to engage an international audience

One of the most humbling accomplishments of CCNS was that our registrants represented every continent (with the obvious exclusion of Antarctica), including many registrants who reached out to say they would not have been able to attend this type of meeting in person. Interestingly, we did not design the meeting with this international audience specifically in mind (indeed, the canceled physical meetings that motivated the creation of CCNS were focused on the Canadian neuroscience community), although we were optimistic that it might naturally arise. The advertising techniques we used organically engaged this type of audience; social media platforms like Twitter and LinkedIn transcend national borders, just as is the oft-cited aspiration of the scientific community at large.

The organizers of future virtual meetings should embrace the possibility that these events might grow beyond their personal locations given the nature of the digital media landscape. This includes considering inviting speakers from locations who might not have been able to travel to an in-person conference and emphasizing resources to minimize the challenges of various time zones (for us, this involved emphasizing the availability of Crowdcast replays). The potential for online meetings to engage academics that may have previously been overlooked by in-person conferences may represent the most direct evidence for the democratizing power of these meetings and should be embraced as much as possible.

However, it bears mentioning that time zone differences still remain a challenge for virtual conferences. While the ability to view talks on replay is a clear improvement over missing them entirely, the replays still lack the level of interactivity of live sessions. While it is impossible to schedule talks in a manner that is perfectly accessible across the globe, one solution that has been implemented [3] is scheduling talks outside of the traditional “9-to-5” day. Given the amount of international engagement garnered by CCNS, we plan to improve its accessibility to the international audience by doing something in this vein in the next iteration of our meeting. Another challenge is that sufficient internet connectivity to view live content is not ubiquitous, although the ability to view talks on replay (which is more easily done with weaker internet connectivity) is one small step toward addressing this gap.

## Rule 8: Ensure your meeting addresses a need unmet by current conferences and fills this gap in an engaging and creative fashion

While organizing a 2-day virtual conference in 10 weeks was a daunting task, it allowed us to fully exploit the benefits of the online setting to create a unique meeting. Specifically, we wanted our meeting to be focused on engaging and highlighting trainees in ways that are typically not the norm at legacy neuroscience conferences. We did this in 3 main ways: First, we began each themed session with a “tutorial” talk to provide an introduction to the field accessible to trainees at various levels and with various backgrounds; second, we held a panel discussion each day with our speakers in which trainees could ask questions of these speakers on a variety of topics that might not come up in a traditional conference question and answer (Q&A) period; and third, we highlighted the work of trainees by including a designated time in the conference itinerary for their presentations, rather than making these talks appear “secondary” by scheduling them alongside other sessions or at inconvenient times.

It was telling that these elements of the conference were among the most warmly received by our attendees. Of the approximately 40 attendees who participated in anonymous polling at the end of the meeting, more than 90% said that CCNS “met the goal of being a ‘trainee-focused’ meeting,” all found the panel discussions at least partially helpful, and all found that the tutorial discussions “benefited the conference.” We think that this led our meeting to have a unique feel from traditional conferences even beyond the new, virtual setting. This success shows that there is an appetite among the academic community for meetings that eschew the traditional conference template in favor of novel strategies for improving this experience.

## Rule 9: Search for novel solutions to facilitate networking opportunities

Part of the trainee-focused meeting was an attempt to design a new type of talk for trainees that provided a greater spotlight while maintaining the interactivity of a traditional poster session. This was the primary area of the meeting that we plan on improving in future iterations, as trying to strike this “middle ground” between a talk and a poster session had clear flaws.

This appears to be a symptom of a more broad challenge facing online meetings: replicating the organic interactions, discussions, and collaborations that arise at in-person meetings [7]. Many of these occur outside of the conference proper in conversations over coffee or drinks facilitated by the critical mass of similarly minded scientists in the same location. The onus is on the organizers of future online meetings to find creative solutions for mimicking these types of interactions in the virtual setting, with the best potentially defining the conference landscape for years to come. For example, there has been considerable recent interest in emerging technologies that combine “explorable” computer role-playing game (CRPG)–type virtual physical environments with video-conferencing functionality, allowing naturalistic conversation between attendees when their avatars are nearby in the virtual space. Given the scientific community’s capacity and appetite for technical challenges and tool development and the manifested need for new solutions to this problem, it seems reasonable to expect major developments in this area in the coming years.

## Rule 10: Craft a coherent, themed conference itinerary to make the content accessible to as broad an audience as possible

An additional element of our conference that made it more accessible both to trainees and scientists with a variety of backgrounds was our early decision to build our meeting around 4 content “themes.” This involved our brief (20 minutes) “tutorials” to introduce a key concept to trainees, multiple “research talks” (30 minutes) building on and applying that concept, and “keynote addresses” (1 hour) going into a specific topic in the field in more detail. These sessions are briefly summarized below to illustrate how we crafted an itinerary that gradually built upon the topics introduced in each session, creating a “flow” that was more easily accessible to a diverse audience.

### Microcircuit oscillations

How neural microcircuits generate the oscillatory behavior thought to play a role in a variety of cognitive processes [8] is a pivotal question in computational neuroscience. An introduction to key mathematical terminology used in this field, specifically bi- and multi-stability [9,10], served to inform talks from Drs. Sue Ann Campbell [11] and Frances Skinner [12] utilizing these tools. This was followed by a keynote presentation from Dr. Nancy Kopell [13], who discussed 3 “stories” of how her work in computational neuroscience has yielded insights into the relationship between the generation of neural oscillations and their functional consequences.

### Macro-level brain oscillations

Meso- and macro-level models of neural dynamics aim to develop coarse-grained descriptions of activity in local neural populations. A didactic introduction to key concepts and formulations used in neural population modeling (neural mass, neural field, and mean-field) [14] began this session. Dr. Jérémie Lefebvre then presented new work modeling the influence of slow adaptive myelination mechanisms on oscillatory whole-brain networks [15]. Finally, Dr. Dimitris Pinotsis’ keynote presentation discussed new directions in the understanding of predictive coding and its implementation in the brain through the laminar and hierarchical structure of oscillatory microcircuit motifs in the cortex [16].

### Neuromodulation

Computational modeling is accelerating our understanding of the mechanisms underlying the effects of neuromodulation. An overview of different neurostimulation techniques and multi-scale brain modeling provided an introduction for this themed session. The therapeutic implication of deep brain stimulation (DBS) as an experimental therapy for psychiatric disorders was the focus of the keynote presentation from Dr. Cameron McIntyre, including an interactive holographic visualization tool [17]. In the subsequent talks, the impact of DBS on modulating information processing was discussed by Dr. Milad Lankarany [18], and applications of DBS in implantable devices were discussed by Dr. Taufik Valiante [19].

### Computational psychiatry

Computational psychiatry broadly investigates whether computational models can address clinical questions in psychiatry, while also unmasking key pathophysiological mechanisms. The aim of this session was to provide an overview of the modeling efforts to make individual predictions in psychiatry, with a particular focus on psychosis [20]. An introduction to hierarchical Bayesian models [21] provided a basis for talks from Drs. Andreea Diaconescu [22] and Maxwell Ramstead [23]. This was followed by a keynote presentation from Dr. Philip Corlett, who presented his recent research examining paranoia in terms of abnormal learning in the presence of uncertainty [24].

## Conclusions

Even before the COVID-19 pandemic, there was growing momentum for a shift away from traditional conferences, given the significant carbon footprint of these meetings [25]. At the time of writing this article, and arguably for the foreseeable future, the pandemic has made this change an immediate necessity. We believe that the success of CCNS can be seen as a “proof of concept” that such online meetings can be free, open, and organized by early career researchers without significant institutional support, while filling unique niches in the existing landscape of academic conferences. The feedback we have received from our attendees has been immensely gratifying, including the fact that everyone surveyed after the conference indicated that they would like to see the meeting return in the future.

It is our hope that the “10 simple rules” we distilled from our personal experience creating CCNS will serve not only to improve future meetings and encourage the formation of new ones (a key element of the “democratization” of the conference landscape), but also to provide evidence that the benefits of online meetings can and should be passed on to attendees. This element of conference democratization arises naturally from a variety of the features of a virtual meeting highlighted in our “rules,” including the ability to easily make content available for replay and include speakers who might not traditionally be spotlighted at physical meetings (due to either travel limitations or the relative lack of emphasis on trainee work). Perhaps the most important way in which this democratization can be achieved is via significantly reduced (if not eliminated) costs of attendance. On this note, it is concerning to hear of many conferences that are moving into the online setting, but not making corresponding adjustments to their fees. The academic community would be remiss if it did not seize this opportunity to make science more accessible via a significant reduction in the costs of conference attendance.
